# The Risk Factors of Severe Hypoglycemia in Older Patients with Dementia and Type 2 Diabetes Mellitus

**DOI:** 10.3390/jpm12010067

**Published:** 2022-01-07

**Authors:** Nai-Ching Chen, Chien-Liang Chen, Feng-Chih Shen

**Affiliations:** 1 Department of Neurology, Kaohsiung Chang Gung Memorial Hospital and Chang Gung University College of Medicine, Kaohsiung 833, Taiwan; naiging@yahoo.com.tw; 2 Department of Medical Education and Research, Kaohsiung Veterans General Hospital, Kaohsiung 813, Taiwan; cclchen@seed.net.tw; 3 Division of Nephrology, Kaohsiung Veterans General Hospital, Kaohsiung 813, Taiwan; 4 Department of Medicine, School of Medicine, National Yang-Ming University, Taipei 112, Taiwan; 5 Division of Endocrinology and Metabolism, Department of Internal Medicine, Kaohsiung Chang Gung Memorial Hospital and Chang Gung University College of Medicine, Kaohsiung 833, Taiwan; 6 Center for Mitochondrial Research and Medicine, Kaohsiung Chang Gung Memorial Hospital, Kaohsiung 833, Taiwan

**Keywords:** Alzheimer’s dementia, hypoglycemia, diabetes, antidiabetic medications

## Abstract

Background: The adequate glycemic control and risk factors for hypoglycemia in older patients with dementia and type 2 diabetes mellitus (T2DM) remain unclear. This study aimed to analyze the status of glycemic control and determine the risk of hypoglycemia among these groups. Methods: A hospital admission record due to hypoglycemia through an emergency room with glucose supplementation in the Chang Gung Memorial Hospital was identified as a hypoglycemic event. Patients with dementia and T2DM without hypoglycemic events throughout the study period were defined as the control group. We gathered patients aged ≥65 years with a diagnosis of Alzheimer’s dementia (AD) and T2DM between 2001 and 2018 in the Chang Gung Research Database (CGRD). We extracted data included medication use, diagnoses, and biochemistry data from hospital records. Results: A total of 3877 older patients with dementia and T2DM with regular visits to the outpatient department were enrolled in this study. During the two-year follow-up period, 494 participants (12.7%) experienced hypoglycemia. Multivariable logistic multivariable regression models for hypoglycemic events showed that metformin had a protective effect (odds ratio (OR) = 0.75, *p* = 0.023), insulin had the highest risk (OR = 4.64, *p* < 0.001). Hemoglobin A1c (HbA1c) levels were not correlated with hypoglycemic events (OR = 0.95, *p* = 0.140). Patients with hypoglycemic episodes had a significantly higher proportion of ≥2 Charlson Comorbidity Index scores than those without hypoglycemic episodes (83.2% versus 56.4%, *p* < 0.001). Conclusions: Drug regimen affects hypoglycemic episodes but not HbA1c in older patients with dementia and T2DM. In addition, patients with more comorbidities experience an increased risk of hypoglycemia.

## 1. Introduction

Dementia is characterized by poor cognitive performance, primarily due to old age, with age-standardized prevalence for those >60 years old, varying from 5.0–8.5% [1,2]. With the progression of an aging population, dementia prevalence increased rapidly, with approximately 65.7 million in 2030 [3]. Furthermore, with changing lifestyle factors, such as diet and overweight, the prevalence of type 2 diabetes mellitus (T2DM) also increases [4]. T2DM may also be associated with adverse vascular risk factors, including obesity, hypertension, and dyslipidemia, which may contribute to the incidence of dementia [4,5].

Poor glycemic control is associated with microvascular or macrovascular complications, and intensive glycemic control, in contrast, could minimize the development of these complications [6,7]. Clinical guidelines recommend targeting a hemoglobin A1c (HbA1c) level of <7.0% for most adults with T2DM [8]. However, patients with complex health problems, limited life expectancy, and advanced age are unlikely to benefit from strict glycemic control. The American Diabetes Association recommended targeting an HbA1c level to less stringent levels (such as HbA1c < 8.0–8.5%) in older adults with T2DM who have multiple coexisting chronic illnesses, cognitive impairment, or functional dependence [9].

Dementia and cognitive function changes would generate a decreased perception of symptoms associated with hypoglycemia and subsequently increase the risk of unawareness or severe episodes [9,10]. These consequences would be associated with an increased hospital stay and mortality, with further cognitive impairment that directly affects the independence and functionality of patients with dementia [9,10].

Previous studies examining the trends in use, effects (glycemic control as measured by HbA1c), and hypoglycemia of glucose-lowering medications have predominantly focused on the older population with T2DM [11,12,13,14]. However, there are limited studies regarding the appropriate glycemic control and risk factors of hypoglycemia in patients with dementia and T2DM. Thus, this study aimed to investigate the risk factors of hypoglycemia among patients’ characteristics and glucose-lowering medications usage in older patients with concomitant T2DM and dementia.

## 2. Materials and Methods

### 2.1. Data Source

The Chang Gung Medical Foundation (CGMF) is the largest medical system in Taiwan. Electronic medical records derived from Chang Gung Memorial Hospital (CGMH) to comprise the Chang Gung Research Database (CGRD) were used to provide real-world evidence and improve clinical and policy decisions [15]. To ensure data privacy, patient and provider information were encrypted and deidentified. This study was conducted according to the guideline of Declaration of Helsinki and Good Clinical Practice and approved by the Institutional Review Board (IRB) of CGMH (approval number 202001795B0, date of approval: 06 January 2021). Informed consent was waived according to IRB regulations.

### 2.2. Selection of Patients with AD and T2DM

This retrospective cohort study used registered data in CGRD from 1 January 2001 to 30 June 2018. First, patients aged ≥65 years who had Alzheimer’s dementia (AD) and regular follow-ups ≥2 years were selected. The diagnosis of AD was based on International Class of Disease, Ninth Revision, Clinical Modification codes (ICD-9-CM codes) (290.0−290.3 and 331.0) and patients undergoing treatment with one of the following drugs: donepezil, rivastigmine, or galantamine. In Taiwan, the diagnosis of AD can only be established by certified psychologists or well-trained neurologists based on strict reviews of medical records, brain images, and blood and cognitive test results. After receiving certification permission, patients were eligible to receive the four aforementioned medications [16].

Secondly, the selected participates with concomitant T2DM were recruited. The diagnosis of T2DM ((ICD-9) codes 250.X) made during the follow-up study period required meeting one of the following criteria: >1 inpatient admission with the diagnosis of T2DM, and with a prescription of antidiabetic medications for >30 days; >3 outpatient visits with a diagnosis of T2DM, with a prescription of antidiabetic medications for >30 days and with regular follow up visits ≥2 years.

We excluded patients with type 1 DM, those with <1 year of continuous follow up visits in outpatient clinic of the CGMH, a history of benign neoplasm of the pancreas, malignant neoplasm of islets of Langerhans, or a missing HbA1c record in CGRD medical records. We considered these exclusion criteria to identify the incidents of hypoglycemia and to avoid capturing hypoglycemic events from pancreas-related cancers.

### 2.3. Definition of Hypoglycemic Event

Hypoglycemia was defined based on the ICD-9 codes 251.0, 251.1, 251.2, and 250.8x (excluding 259.8, 272.7, 681.xx, 682.xx, 686.9x, 707.xx, 709.3, 730.0, 730.1,730.2, and 731.8, which represent other diseases, such as cellulitis and lower extremity ulcers). The study participates who had their emergency department (ED) visit with a diagnosis of hypoglycemia and received the prescription of 50% glucose solutions in the ED further confirmed the hypoglycemic events.

### 2.4. Definition and Measurement of Biochemical and Medical Data

In hypoglycemia group, the laboratory data within 3 days of the date of hypoglycemic events (index date) were collected for analysis. Additionally, the prescription of glucose-lowering medications within 28 days before the index date was recorded. In patients without a hypoglycemia event, we collected the record of laboratory data and glucose-lowering medications from the last outpatient clinic visit in the study periods.

The values of HbA1c, total cholesterol, low-density lipoprotein (LDL), serum creatinine, and estimated glomerular filtration rate (e-GFR) were obtained from the clinical laboratory system at the same day of laboratory test. Data sources were linked using the unique and permanent CGRD identification number.

To evaluate patient comorbidity, we used the diagnosis from medical charts to calculate the Charlson Comorbidity Index (CCI) [17,18], which is a measurement of comorbidity burden and has been correlated with life expectancy.

### 2.5. Statistical Analysis

Continuous data are expressed as means with standard deviation, and categorical data are expressed as numbers and percentages. Power was larger than 90%, and it was calculated by logistic regression from CCI and hypoglycemic event. We use univariable logistic regression analysis to access the contributing factors for hypoglycemia. Then, those statistically significant factors in univariable logistic regression analysis were enrolled into binary logistic multivariable regression analysis to identify the independent risk factors for hypoglycemia in older patients with concomitant T2DM and dementia. To avoid the interaction effect, we choose creatinine rather than e-GFR and lab data rather than comorbidities. The data were managed and cleaned using SAS software (version 9.4; SAS Institute Inc., Cary, NC, USA).

## 3. Results

### 3.1. Study Process Flowchart

**Figure 1:**
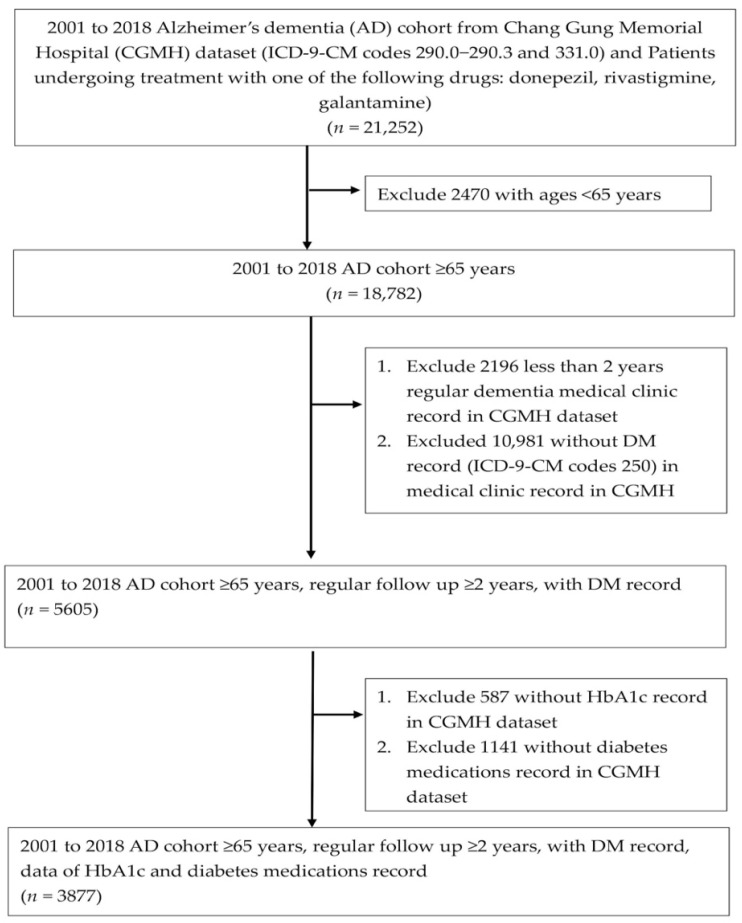
Study process flow chart. ICD-9-CM, International Class of Disease, Ninth Revision, Clinical Modification.

Figure 1 shows the flowchart for the selection of the study subjects. Data from 3877 patients with T2DM and AD (1454 men and 2423 women) were analyzed. Finally, there were 494 patients with T2DM and AD who had hypoglycemic episodes.

### 3.2. Patient Characteristics and Comorbidities

The median age was 77.5 years old, and 62.5% of the patients were females (Table 1). A total of 59.8% of the patients had a >2 CCI. Moreover, the mean HbA1c was 7.04%, and the proportion of HbA1c ≤ 7% was 60.6% among the study patients. Approximately one-third of the patients had chronic kidney disease, which was defined as an estimated glomerular filtration rate (e-GFR) ≤ 60 mL/min/1.73 m^2^.

Renal disease was the most common comorbidity (36.9%), followed by cerebral vascular accident (34.9%), peptic ulcer disease (24%), and pulmonary disease (16.6%). Metformin was the most commonly used antidiabetic medication (36.9%), followed by sulfonylureas (32.2%), insulin (31.1%), and DPP-4 inhibitors (27.9%).

### 3.3. Different Characteristics and Comorbidities between Patients with and without Hypoglycemic Events

There was no statistically significant difference in sex (*p* = 0.307) and age (*p* = 0.122) between patients with and without hypoglycemia (Table 2). In addition, patients with hypoglycemic events had lower LDL (*p* < 0.001), lower total cholesterol (*p* < 0.001), and higher creatinine levels (*p* < 0.001) than those without hypoglycemic events. However, HbA1c levels had no changing effect between patients with and without hypoglycemic events.

Patients with hypoglycemic episodes had more acute myocardial infarction, congestive heart failure, peripheral vascular disease, cerebral vascular accident, pulmonary disease, peptic ulcer, liver disease, renal disease, and cancer (all *p* < 0.05) (Table 2).

### 3.4. The Usage of Antidiabetic Medications in Patients with and without Hypoglycemic Event

Patients who suffered from a hypoglycemic event received a lower percentage of metformin than those who did not (*p* < 0.001) (Table 2). Moreover, they had more prescriptions of insulin (62.8%), sulfonylureas (36.4%), α-glucosidase inhibitors (17.2%), thiazolidinediones (8.5%), and meglitinides (10.3%) than those without hypoglycemic events (all *p* < 0.05).

### 3.5. Risk Factors Correlated with Hypoglycemic Events in Patients with AD and T2DM

In univariable logistic regression analysis (Table 3), the patients who had CCI ≥ 2 had higher risk of hypoglycemic episodes (Crude Odds ratio = 6.59, *p* < 0.001). In addition, higher LDL (Crude Odds ratio = 0.50, *p* < 0.001) and total cholesterol (Crude Odds ratio = 0.49, *p* = 0.002) had a protective effect from a hypoglycemic event in patients with AD and T2DM. Normal renal function included normal creatinine or e-GFR > 60 had a protective effect from a hypoglycemic event in patients with AD and T2DM.

Patients who received metformin treatment had a protective effect against hypoglycemia (adjusted odds ratio (aOR) = 0.75, *p* = 0.023) compared to those without metformin treatment under multivariable logistic regression test (Table 4). Insulin (aOR = 4.64, *p* < 0.001), thiazolidinediones (aOR = 2.04, *p* = 0.0004), combinations of oral blood glucose lowering drugs (aOR = 1.92, *p* < 0.0001), and sulfonylureas (aOR = 1.46, *p* = 0.0007); however, these patients were more likely to induce hypoglycemic events (Table 3). Moreover, the higher the CCI, the higher the risk of hypoglycemia. Those with higher CCI (≥2) had a higher risk of hypoglycemic events (aOR = 6.59, *p* < 0.001) than those without CCI (Table 3). The occurrence rate of hypoglycemia was similar across different HbA1c levels (Table 5).

## 4. Discussion

In this study, we confirmed the risk factors of severe hypoglycemic episodes in patients with dementia and T2DM. We also found that drug regimens, especially insulin and sulfonylurea, were associated with an increased risk of hypoglycemia in older patients with AD and T2DM. However, metformin had a protective effect against hypoglycemia. In older adult patients with T2DM, we did not observe a U-shaped relationship, such that the lowest and highest glucose levels were associated with an increased risk of hypoglycemia.

Moreover, about 12.7% of participants with concomitant AD and T2DM experienced of hypoglycemic event in our study. In Thailand, an analysis on the DM dataset in 2014 with a nationwide survey showed only 3.1% severe hypoglycemic events in older individuals >65 years old [19]. However, a similar study with the included population (60–75 years old) in different reports also had a higher percentage (10.6%) [20]. Thus, older patients with DM and AD have been recognized to have a higher risk of developing hypoglycemia [19,21,22]. In addition, a higher risk of severe hypoglycemia occurred in older patients with DM and AD because these populations are less able to recognize hypoglycemia and avoid hypoglycemia by initiating appropriate responses. Furthermore, these patients are likely to take accidental drug overdoses or skip meals, resulting in recurring events of hypoglycemia. [23,24]

The number of comorbidities is significant and correlated with hypoglycemic events based on managing patients with AD and T2DM. The patients with hypoglycemic events had ≥2 CCI. The OR was 6.59 in patients with >2 CCI, and it was the highest among all risk factors. The trends of severe hypoglycemia are associated with multiple comorbidities [25,26]. In addition to the number of comorbidities, a single disease is also associated with hypoglycemic events. In our study, patients with peptic ulcer and renal disease had a higher percentage of hypoglycemic events. A total of 11,404 older T2DM patients in Thailand in 2014 also revealed that cardiovascular disease and peripheral artery disease were associated with severe hypoglycemic events [19].

Glucose-lowering medications play an important role in the occurrence of hypoglycemia. Thus, pharmacologic regimens need to be adjusted to minimize the development of hypoglycemia. Metformin, a widely accepted first-line therapy for T2DM, is characterized by good glucose-lowering effects and a lower risk of hypoglycemia. Indeed, the incidence of hypoglycemia was lower in patients using metformin than in those without metformin in our study. Our results support the current recommendation for glucose-lowering treatment for older adults [9,27].

In contrast to metformin, the use of sulfonylureas or glinides was associated with a higher risk of hypoglycemia. The insulin-releasing action of these drugs can be persistent at low glucose levels, which would lead to the development of hypoglycemia, making these drugs an undesirable choice for older patients with diabetes [28]. Furthermore, the OR of hypoglycemia events related to insulin prescription was 4.68 among all glucose-lowering drugs. In clinical practice, insulin timing therapy is usually initiated when oral glucose-lowering drugs do not provide adequate glycemic control. However, patients would have more comorbidities or DM-related complications when insulin is started, which could result in a high probability of hypoglycemia [25,26,29]. Thus, these classes of glucose-lowering drugs should be used carefully in older patients with T2DM [27].

HbA1c levels did not correlate with hypoglycemic episodes. There is a growing consensus regarding increased risk for hypoglycemia and reduced potential to benefit from tight glycemic control; older patients with DM and AD should avoid tight glycemic control (HbA1c < 7%) and instead pursue “safe glycemic range” HbA1c levels < 8% due to coexisting severe medical conditions, the presence of cognitive dysfunction, and ability to perform daily activities [30,31,32]. Moreover, the population reached HbA1c levels (7.1%), as indicated by international recommendations, patients with AD had a significantly higher prevalence of hypoglycemia than those without AD [31,32]. This requires an investigation to test if such recommendations are suitable for patients with AD and identify the antidiabetic agents that provide the safest profile.

This study proved that the CGRD provides more clinical information, particularly for lab data results, which is unavailable in the National Health Insurance Research Database in Taiwan. Therefore, this allowed us to examine the possible factors of hypoglycemic events in large sample size of patients with AD and T2DM. In Taiwan, the diagnosis of AD can only be established by certified psychologists or well-trained neurologists based on strict reviews of medical records, brain images, and blood and cognitive test results. After receiving certification permission, the patients were eligible to receive acetylcholinesterase inhibitors for treatment. Therefore, the validation of the accurate diagnosis of AD is better than other claim datasets, which are mainly based on ICD-9-CM or the diagnostic and statistical manual of mental disorders criteria.

This study had several limitations. First, the percentage of severe hypoglycemia might be higher when the patients visited other hospitals for hypoglycemic events, and their medical information in CGRD will not be completed. Second, the patients’ complete comorbidities data from the CGRD may be lower than those from the national database because the patients might have visited other hospitals for different reasons. Third, we only collected data on hypoglycemic episodes, associated medications, laboratory data, and comorbidities and not on the patients who suffered from several episodes of hypoglycemic events. Fourth, patients with AD often have other risk factors for hypoglycemia, including weight loss, changes in appetite, and eating habits. Thus, this retrospective database cannot provide a detailed analysis.

## 5. Conclusions

In summary, the results show more than two comorbidities of T2DM in older patients with comorbid AD, potentially placing them at an increased risk for severe hypoglycemia and serious adverse effects. In addition, our results suggest that insulin or sulfonylureas should be used with caution in older patients with T2DM and dementia, while the metformin seems to be safer. The present findings add insight for future studies testing prospective severe hypoglycemic events in older patients with concomitant T2DM and dementia. Further studies investigating the use of different classes of glucose-lowering medications on changes in cognitive abilities or impact on quality of life in older patients with diabetes and dementia will provide substantial evidence for their use.

## Figures and Tables

**Table 1:** Patient characteristics for all patients with Alzheimer’s dementia and type 2 diabetes.

Variables	*n*, (%)
Patients, number	3877
Gender	
Male	1454 (37.5%)
Female	2423 (62.5%)
Age (years)	77.5 (8.9)
Charlson Comorbidity Index	
0	664 (17.1%)
1	893 (23.0%)
≥2	2320 (59.9%)
HbA1c (%)	7.04 ± 1.36
HbA1c (%)	
value ≤ 7	2350 (60.6%)
7 < value ≤ 9	1235 (31.9%)
value > 9	292 (7.5%)
LDL (mg/dL)	90.14 ± 30.70
LDL (mg/dL)	
Not available	889 (22.9%)
<70	748 (19.3%)
70–100	1276 (32.9%)
≥100	964 (24.9%)
Total cholesterol (mg/dL)	156.99 ± 34.23
Total cholesterol (mg/dL)	
Not available	836 (21.6%)
<200	2625 (67.7%)
≥200	416 (10.7%)
Creatinine (mg/dL)	1.42 ± 1.41
Creatinine (normal range men: 0.57–1.02, women: 0.68–1.19) (mg/dL)	
Not available	486 (12.5%)
Within range	1462 (37.7%)
Above range	1510 (38.9%)
Under range	419 (10.8%)
e-GFR (mL/min/1,73 m^2^)	66.64 ± 39.18
e-GFR (mL/min/1,73 m^2^)	
Not available	486 (12.5%)
≤60	1432 (36.9%)
>60	1959 (50.5%)
Systemic disease	*n* (%)
Acute myocardial infarction	199 (5.1%)
Congestive heart failure	410 (10.6%)
Peripheral vascular disease	90 (2.3%)
Cerebral vascular accident	1352 (34.9%)
Pulmonary disease	642 (16.6%)
Peptic ulcer	931 (24.0%)
Liver disease	416 (10.7%)
Paraplegia	79 (2.0%)
Renal disease	1432 (36.9%)
Cancer	489 (12.6%)
Antidiabetic medication	*n* (%)
Metformin	1474 (38.0%)
Insulin	1205 (31.1%)
Sulfonylureas	1250 (32.2%)
α-glucosidases inhibitors	525 (13.5%)
Thiazolidinediones	198 (5.1%)
DPP-4 inhibitors	1081 (27.9%)
GLP-1 receptor agonists	19 (0.5%)
SGLT2 inhibitors	67 (1.7%)
Meglitinides	304 (7.8%)
Combinations of oral blood glucose lowering drugs	1105 (28.5%)

Values are displayed as *n* (%), mean ± standard deviation, or mean (interquartile range). For hemoglobin and creatinine, the reference values are displayed for males and females separately. LDL, low density lipoprotein; e-GFR, estimated glomerular filtration rate; DPP-4, dipeptidyl peptidase-4; GLP-1, glucagon-like peptide 1; SGLT2, sodium glucose cotransporter 2.

**Table 2:** The difference of clinical characteristics, antidiabetic medications, and comorbidities among patients with and without hypoglycemic events.

Variables	Hypoglycemia	No Hypoglycemia	*p* Value
Patients, number	494	3383	
Female	319 (64.6%)	2104 (62.2%)	0.307
Age (years)	77.1 (8.8)	77.6 (8.9)	0.122
Charlson Comorbidity Index			<0.001
0	21 (4.3%)	643 (19%)	
1	62 (12.6%)	831 (24.6%)	
≥2	441 (83.2%)	1909 (56.4%)	
HbA1c (%)	6.96 ± 1.35	7.06 ± 1.36	0.140
LDL (mg/dL)	83.86 ± 30.82	91.42 ± 30.62	<0.001
Total cholesterol (mg/dL)	151.06 ± 38.10	162.47 ± 37.13	<0.001
Creatinine (mg/dL)	1.94 ± 1.89	1.33 ± 1.31	<0.001
Metformin	120 (24.3%)	1354 (40.0%)	<0.001
Insulin	310 (62.8%)	895 (26.5%)	<0.001
Sulfonylureas	180 (36.4%)	1070 (31.6%)	0.033
α-glucosidases inhibitors	85 (17.2%)	440 (13.0%)	0.011
Thiazolidinediones	42 (8.5%)	156 (4.6%)	<0.001
DPP-4 inhibitors	142 (28.7%)	939 (27.8%)	0.647
GLP-1 receptor agonists	1 (0.2%)	18 (0.5%)	0.327
SGLT2 inhibitors	6 (1.2%)	61 (1.8%)	0.348
Meglitinides	51 (10.3%)	253 (7.5%)	0.028
Combinations of oral blood glucose lowering drugs	159 (32.2%)	946 (28.0%)	0.052
Acute myocardial infarction	48 (9.7%)	151 (4.5%)	<0.001
Congestive heart failure	92 (18.6%)	318 (9.4%)	<0.001
Peripheral vascular disease	18 (3.6%)	72 (2.1%)	0.037
Cerebral vascular accident	225 (45.6%)	1127 (33.3%)	<0.001
Pulmonary disease	118 (23.9%))	524 (15.5%)	<0.001
Peptic ulcer	203 (41.1%)	728 (21.5%)	<0.001
Liver disease	74 (15.0%)	342 (10.1%)	0.001
Paraplegia	9 (1.8%)	70 (2.1%)	0.716
Renal disease	281 (56.9%)	1151 (34.0%)	<0.001
Cancer	87 (17.6%)	402 (11.9%)	<0.001

Values are displayed as *n* (%), mean ± standard deviation, or mean (interquartile range). For hemoglobin and creatinine, the reference values are displayed separately for males and females. LDL, low density lipoprotein; e-GFR, estimated glomerular filtration rate. DPP-4, dipeptidyl peptidase-4; GLP-1, glucagon-like peptide 1; SGLT2, sodium glucose cotransporter.

**Table 3:** Univariable logistic regression analysis of clinical characteristics, antidiabetic medications, and comorbidities among patients with and without hypoglycemic events.

Factors	Crude Odds Ratio	95% CI	*p* Value
Gender			
Male	Ref.		
Female	1.11	0.91–1.35	0.307
Age (years)	0.99	0.97–1.00	0.142
Charlson Comorbidity Index			
0	Ref.		
1	2.28	1.38–3.79	0.001
≥2	6.59	4.21–10.31	<0.001
HbA1c (%)	0.95	0.88–1.02	0.140
HbA1c (%)			
value ≤ 7	Ref.		
7 < value ≤ 9	0.92	0.75–1.14	0.454
9 < value	0.90	0.63–1.31	0.591
LDL (mg/dL)			
NA	Ref.		
<70	1.25	0.97–1.61	0.089
70–100	0.82	0.65–1.05	0.113
>100	0.50	0.37–0.67	<0.0001
Total cholesterol (mg/dL)			
NA	Ref.		
<200	0.92	0.75–1.11	0.377
≥200	0.49	0.31–0.77	0.002
Creatinine (normal range men: 0.57–1.02, women: 0.68–1.19) (mg/dL)			
NA	Ref.		
Within range	0.50	0.38–0.65	<0.0001
Above range	1.05	0.84–1.32	0.665
Under range	0.49	0.33–0.73	0.0004
e-GFR (mL/min/1.73 m^2^)			
NA	Ref.		
≤60	1.07	0.86–1.34	0.549
>60	0.46	0.36–0.60	<0.0001
Metformin	0.48	0.39–0.60	<0.0001
Insulin	4.68	3.84–5.71	<0.0001
Sulfonylurea	1.24	1.02–1.51	0.033
α-glucosidases inhibitor	1.39	1.08–1.79	0.011
Thiazolidinedione	1.92	1.35–2.74	0.0003
DPP-4 inhibitors	1.05	0.85–1.29	0.647
GLP-1 receptor agonist	0.38	0.05–2.85	0.346
SGLT2 inhibitor	0.67	0.29–1.56	0.353
Meglitinide	1.43	1.04–1.96	0.028
Combinations of oral blood glucose lowering drugs	1.22	1.00–1.50	0.052
Acute myocardial infarction	2.30	1.64–3.23	<0.0001
Congestive heart failure	2.21	1.71–2.84	<0.0001
Peripheral vascular disease	1.74	1.03–2.94	0.039
Cerebral vascular accident	1.67	1.38–2.03	<0.0001
Pulmonary disease	1.71	1.36–2.15	<0.0001
Peptic ulcer	2.54	2.09–3.10	<0.0001
Liver disease	1.57	1.19–2.05	0.0012
Paraplegia	0.88	0.44–1.77	0.717
Renal disease	2.98	2.46–3.62	<0.0001
Cancer	1.59	1.23–2.04	0.0004

For creatinine, the reference values are displayed separately for males and females. CI, confidence interval; Ref., reference; LDL, low density lipoprotein; NA, not available; e-GFR, estimated glomerular filtration rate; DPP-4, dipeptidyl peptidase-4; GLP-1, glucagon-like peptide 1; SGLT2, sodium glucose cotransporter.

**Table 4:** Multivariable logistic regression model for risk of hypoglycemia in older patients with dementia and type 2 diabetes.

Factors	Adjusted Odds Ratio	95% CI	*p* Value
Charlson Comorbidity Index			
0	Ref.		
1	2.21	1.32–3.71	0.0027
≥2	4.76	2.99–7.56	<0.0001
LDL (mg/dL)			
NA	Ref.		
<70	1.12	0.76–1.67	0.561
70–100	0.84	0.57–1.22	0.353
>100	0.60	0.38–0.95	0.030
Total cholesterol (mg/dL)			
NA	Ref.		
<200	1.28	0.91–1.80	0.161
≥200	0.99	0.54–1.81	0.984
Creatinine (normal range men: 0.57–1.02, women: 0.68–1.19) (mg/dL)			
NA	Ref.		
Within range	0.63	0.46–0.85	0.0023
Above range	0.91	0.69–1.18	0.462
Under range	0.60	0.39–0.91	0.018
Metformin	0.75	0.59–0.96	0.023
Insulin	4.64	3.73–5.78	<0.001
Sulfonylureas	1.46	1.17–1.82	0.0007
α-glucosidases inhibitors	1.24	0.94–1.64	0.132
Thiazolidinediones	2.04	1.37–3.05	0.0004
Meglitinides	1.24	0.88–1.76	0.219
Combinations of oral blood glucose lowering drugs	1.92	1.51–2.45	<0.0001

CI, confidence interval; Ref., reference; LDL, low density lipoprotein; NA, not available.

**Table 5:** Binary logistic regression models for hypoglycemia in different HbA1c quartile among elderly patients with dementia and type 2 diabetes.

HbA1c	Events	Odds Ratio	95% CI	*p* Value
≤5	5 (0.12%)	Ref		
5 < value ≤ 6	112 (2.89%)	1.15	0.44–3.00	0.777
6 < value ≤ 7	191 (4.93%)	0.97	0.37–2.50	0.942
7 < value ≤ 8	106 (2.73%)	0.94	0.36–2.45	0.893
8 < value ≤ 9	45 (1.16%)	0.97	0.36–2.62	0.959
9 < value ≤ 10	19 (0.49%)	1.05	0.37–3.02	0.927
>10	16 (0.41%)	0.96	0.30–3.02	0.941

Values are displayed as *n* (%). CI, confidence interval.
